# Introducing the Concept of Embodied Interconnectedness: Perspectives on Tactile Massage for Adolescents With Attention Deficit Hyperactivity Disorder (adhd)

**DOI:** 10.1177/27536130261475332

**Published:** 2026-08-03

**Authors:** Anna-Carin Robertz, Anne-Katrin Kantzer, Viola Nyman, Carl-Johan Törnhage, Stefan Nilsson

**Affiliations:** 1 Institute of Health and Care Sciences, Sahlgrenska Academy, University of Gothenburg, Gothenburg, Sweden; 2 Child and Adolescent Psychiatry Clinic, 59572 NU Hospital Group, Trollhättan, Sweden; 3 Department of Health Sciences, 42749 University West, Trollhättan, Sweden; 4 Department of Research and Development, NU-Hospital Group, Trollhättan, Sweden; 5 Sahlgrenska Academy, Institution for Clinical Science, University of Gothenburg, Gothenburg, Sweden; 6 Department of Paediatrics, Skaraborg Hospital, Skövde, Sweden; 7 Department of Research and Development, Skaraborg-Hospital Group, Skövde, Sweden; 8 Centre for Person-centred Care (GPCC), Sahlgrenska Academy, University of Gothenburg, Gothenburg, Sweden; 9 Queen Silvia Children’s Hospital, Sahlgrenska University Hospital, Gothenburg, Sweden

**Keywords:** adolescents, attention deficit hyperactivity disorder, complementary therapy, body-mind relations, emotional adjustment, massage therapy

## Abstract

**Introduction:**

Adolescents with attention deficit hyperactivity disorder (adhd) often experience difficulties with attention, impulse control, and emotional regulation, which may limit engagement with traditional therapies. Body-based interventions may offer a complementary approach. Over the past two decades, research on affective touch and C-tactile (CT) fibres has advanced understanding of how gentle, slow stroking contributes to emotional regulation, stress reduction, and social bonding. While these studies demonstrate physiological and psychological effects of CT-targeted touch, translation into everyday clinical practice remains limited. In particular, adolescents’ experiences of such interventions are rarely explored, and those with adhd are underrepresented. This study aimed to illuminate experiences, potential benefits, and acceptability of a tactile massage intervention for adolescents with adhd through a process evaluation including adolescent and guardian perspectives.

**Method:**

Fourteen adolescents aged 15–17 years diagnosed with adhd were recruited from a child and adolescent psychiatry clinic in Sweden and participated in ten weekly sessions of tactile massage. Semi-structured interviews were conducted with adolescents (n = 13) and guardians (n = 12) following the intervention. Qualitative data were analysed using interpretive description, including member checking. Descriptive questionnaire data were collected to contextualise experiences of comfort, relaxation, and feasibility.

**Results:**

Embodied interconnectedness emerged as a central concept describing how tactile massage supported integration of bodily sensations, emotional states, and relational safety. Participants described the intervention as a predictable, respectful, and calming caring encounter. Two experiential patterns were identified: (1) the importance of a structured professional ritual within a safe environment and (2) touch as a pathway to calm and increased bodily awareness. Adolescents reported greater attention to internal bodily signals and increased comfort with physical touch over time.

**Discussion:**

Tactile massage, delivered as a structured affective touch intervention, may support embodied interconnectedness in adolescents with adhd and inform the integration of touch-based approaches into clinical care.

## Highlights

• Embodied interconnectedness developed through integrated bodily, emotional, and relational experiences
• Structured tactile massage fostered safety, trust, and predictability
• Touch supported calm, relaxation, and increased bodily awareness
• Tactile massage was feasible and acceptable for adolescents with adhd
• Embodied interconnectedness shows promise as a conceptual nursing model

## 1. Introduction

Adolescence is a critical developmental period characterised by profound biological, psychological and social transitions, during which identity is formed and behavioural and health patterns are established that can shape wellbeing across the life course.^1^ Adolescents with attention deficit hyperactivity disorder (adhd) often face a complex interplay of cognitive, emotional, and sensory challenges.^2,3^ Beyond the core symptoms of inattention, impulsivity, and hyperactivity, many experience heightened stress reactivity, emotional dysregulation and difficulties in self-regulation. Difficulty with interoception – the ability to sense and interpret internal bodily states – is also common.^4-6^ These factors can limit the adolescents’ ability to engage with and benefit from conventional therapeutic approaches that rely heavily on verbal processing or executive functioning.^7^

Over the past two decades, research on affective touch and C-tactile (CT) afferents, primarily conducted with adults, has substantially advanced understanding of how gentle, slow stroking supports healthy development, emotional regulation, stress reduction, and social bonding.^8,9^ Although some studies have examined these processes in children and adolescents, the evidence base in younger populations remains comparatively limited.^10^ Studies on healthy adults have shown that CT fibres are unmyelinated, low-threshold mechanoreceptive afferents that respond optimally to light-pressure, skin-temperature touch delivered through gentle stroking at velocities of approximately 1–10 cm/s. Sensory information from these fibres is transmitted to the insular cortex, where activation can elicit behavioural, hormonal, and autonomic responses involved in emotional processing and regulation.^5,11-14^ The results of studies on predominantly adult populations further indicate that these responses are associated with reductions in physiological stress markers, such as cortisol, decreased pain perception, increased oxytocin release and prosocial behaviour, enhanced well-being and greater social connectedness.^15-18^

Touch-based interventions, including massage therapy, have long been embedded in nursing practice as part of basic comfort and care, with historical accounts highlighting their role in supporting psychophysiological well-being and compassionate nursing values.^19^ In mental health and broader nursing contexts, touch is described as a meaningful component of compassionate practice, capable of conveying safety, connection, and authenticity when used with sensitivity to individual needs and boundaries.^20,21^ Tactile massage employs gentle, rhythmic strokes applied with moderate pressure, which are thought to activate CT fibres.^22^ Activating these fibres has been associated with decreased stress and pain, as well as enhanced overall well-being.^16^ Previous research in adults has shown benefits, such as lower sympathetic nervous system activity, increased relaxation, and relief from anxiety, sleep issues, depression, and stress.^22-24^ Qualitative studies in adults have shown that tactile massage can promote increased body awareness, self-confidence, and a sense of wholeness. Participants have described experiences of togetherness, trust, and emotional safety when receiving undivided attention in a therapeutic relationship with a nurse.^25-27^ A more recent systematic review found potential benefits of deep tissue massage for children and adolescents, including reduced adhd symptoms, anxiety, and asocial behaviour, although methodological limitations warrant caution.^28^ In Sweden, tactile massage has been used as a complementary intervention for adolescents with adhd; however, data on tactile massage in this population are limited and robust scientific evidence supporting its effectiveness is currently lacking.^29^

While a few studies have demonstrated the physiological and psychological effects of CT-targeted touch, its translation into routine clinical practice, including its applicability and acceptability among adolescents, remains limited.^8,16^ Existing research involving individuals under 18 years of age has primarily focused on autism spectrum conditions, suggesting potential benefits for emotional well-being, regulation, and social engagement.^8,9^ Consequently, little is known about how adolescents with adhd experience CT-targeted touch therapy or how such interventions can be implemented in this population. Given the limited representation of adolescents with adhd in the existing literature, this remains an important gap in current knowledge.

Research on tactile interventions for adolescents with adhd is limited, especially studies that examine both physiological and psychological outcomes. While strong evidence supports the therapeutic value of touch, further research is needed to understand how it can be effectively implemented in practice. Moreover, touch therapy is inconsistently theorised and variably accepted within professional nursing discourse,^20,21^ which may contribute to its limited implementation. Within contemporary nursing science, there appears to be both a conceptual and clinical gap regarding touch therapy. Previous nursing concepts do not fully account for the embodied and sensory experiences emerging through tactile massage. They emphasise wholeness and relational care,^30^ and although they provide important theoretical insights, they do not fully capture the directly embodied and non-verbal dimensions of tactile care. This study seeks to address that gap by building on the earlier efficacy study (Study 1) for the TaMa-ADHD project.^31^ This study aimed to illuminate the experiences, potential benefits, and acceptability of a tactile massage intervention for adolescents with adhd using a process evaluation that included both adolescent and guardian perspectives.

## 2. Methods

### 2.1. Design

#### 2.1.1. Overview of Study 1

Study 1 was conducted as a small-scale quantitative impact study within the TaMa-ADHD project and explored preliminary effects of tactile massage for adolescents with adhd. Study 1 was conducted as a quantitative impact study and examined the impact of the intervention on outcome measures. Data for Study 1 were collected at five time points before the intervention (T0) in January 2021, during (T1–T3), and at follow-up (T4) in September 2021. During the massage sessions (between February and June), data were collected after the first massage session (T1), after the sixth session (T2), and after the tenth session (T3). These assessment points were consistent for all participants (labelled A-N), except for participant M, who received six massage sessions and therefore had missing data at T3, and participant N, who received three massage sessions and consequently had missing data at both T2 and T3. The results from this study indicated that tactile massage may reduce core adhd symptoms, improve sleep, and lessen pain interference, as measured through validated questionnaires and a single-subject ABA design.^23^

#### 2.1.2. Participants

Participants were recruited from a child and adolescent psychiatric clinic located in western Sweden and were the same for Study 1 and Study 2. Inclusion criteria were a confirmed adhd diagnosis (F90.0B), being either unmedicated or on a stable regimen of central stimulants and/or atomoxetine for the past six months, and fluency in Swedish. Exclusion criteria comprised severe psychiatric comorbidities, suicidal ideation, substance abuse, mental impairment, or current participation in other psychological treatments. Initially, 377 adolescents aged 15 to 17 with adhd and comorbid conditions were identified through review of medical records. From this pool, a more uniform subgroup of 195 individuals with combined type adhd and none of the exclusionary diagnoses was selected. Seventy-eight adolescents were deemed potential participants. A statistician, not involved in the study, randomised these individuals into five groups based on age and gender to ensure group comparability. Recruitment involved providing written information and follow-up via telephone, concluding when 14 adolescents agreed to participate. Consent was also secured for separate questionnaires and post-intervention interviews from their guardians, with 13 agreeing to participate. The final sample consisted of 14 adolescents (five girls and nine boys), who were all outpatients. In line with qualitative process evaluation methodology, sample size was guided by information power rather than statistical representativeness. The participants had an average age of 16.4 years and had been diagnosed with adhd for an average of 3.6 years before participating in the study. All adolescents who fulfilled the inclusion criteria were asked to complete the adhd section of the Mini International Neuropsychiatric Interview for Children and Adolescents (MINI-KID),^32^ a structured diagnostic tool commonly used in psychiatric assessments for individuals up to 18 years of age. The massage intervention was carried out in two cities within the service area of the child and adolescent psychiatric clinic from February to June 2021. For baseline characteristics of the participants, see Table 1.

**Table 1.** Baseline Characteristics of Participants: Age, Sex, Diagnosis, Duration and Medication

Patient label	Age at baseline (years)	Sex	Duration adhd Diagnosis(years)	Baseline medication (T0)
A	16	F	3	Lisdexamfetamine + Esomeprazole
B	16	M	8	Atomoxetine + Melatonin
C	15	F	4	Methylphenidate + Melatonin + Promethazine
D	17	M	7	Lisdexamfetamine + Promethazine
E	15	F	3	Methylphenidate
F	17	M	5	None
G	15	M	1	Methylphenidate
H	16	M	5	Dexamphetamine
I	17	M	3	Methylphenidate
J	16	F	1	None
K	15	M	10	Methylphenidate
L	16	F	6	Lisdexamfetamine
M	16	M	7	None
N	15	M	1	Lisdexamfetamine + Melatonin

*Note.* F = female, M = male.

#### 2.1.3. Tactile Massage Intervention

The intervention consisted of ten weekly sessions of tactile massage focused on the back, arms, hands and feet. To ensure intervention fidelity, all study intervention providers had previously completed TaktiPro® training and certification, received additional study-specific training, and were continuously monitored to ensure adherence to the intervention protocol. This standardised approach ensured that all adolescents received equivalent treatment. A total of 129 tactile massage sessions were delivered by seven female certified massage providers. Certification as a TaktiPro® provider includes two days of theoretical and practical training, followed by 60 hours of supervised hands-on practice and successful completion of both theoretical and practical examinations. The certified massage providers were external to the Child and Adolescent Clinic and represented a range of professional backgrounds. Two were registered nurses (RNs), two were assistant nurses, one was a deacon, one a primary school teacher, and one a behavioural scientist. At the outset of the study, the intention was for each adolescent to receive all massage sessions from the same massage provider. However, due to variations in massage provider availability and capacity, the number of sessions conducted by each ranged from 8 to 34. In addition, one massage provider withdrew from the study during the intervention period. As a result, three participants received all massage sessions from the same massage provider, while the remaining participants received sessions from two to four different massage providers. The complete massage protocol is provided in Supplementary File S1.

#### 2.1.4. Hybrid Design

**Figure 1.**
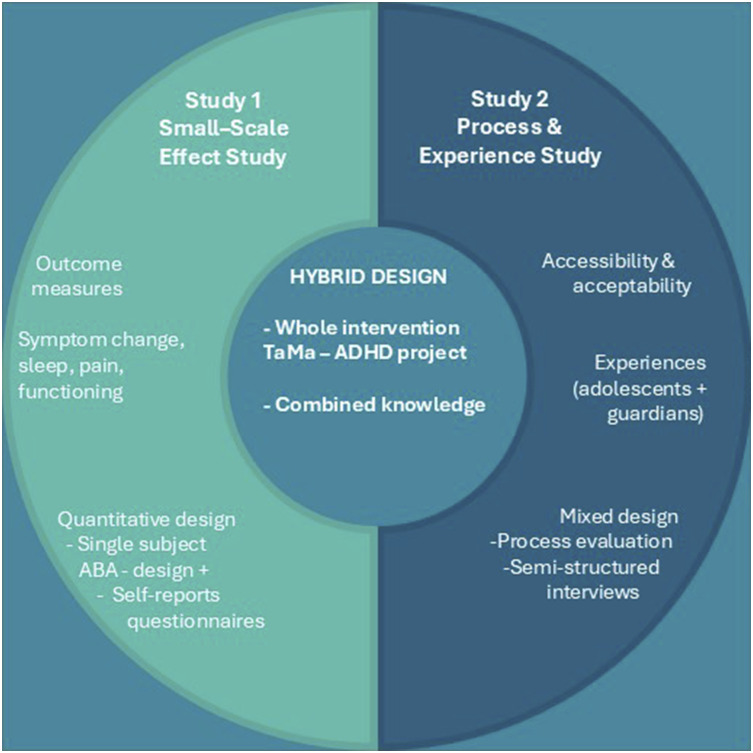
Study 1 contributes early indications of potential effects (“What happened?”), while Study 2 explains processes, meaning, and feasibility (“How and why did it happen?”). Together they form a coherent whole

Study 1 Ref. ^23^ and Study 2 (the current study) are linked through a hybrid design^24,25^ that combines different methodological approaches while involving the same participant sample. Building on the findings from Study 1, Study 2 integrates qualitative and descriptive quantitative methods to deepen understanding of participants’ experiences, as well as the acceptability, feasibility, and perceived meaning of the intervention. This coherent design ensures that both studies are grounded in the actual needs and experiences of the adolescents and their guardians. Together, the studies aimed to generate clinically relevant knowledge informed by both measurable outcomes and lived experiences. The relationship between the studies is illustrated in Figure 1.

#### 2.1.5. Study Design for Study 2

##### 2.1.5.1. Research questions

• What were adolescents’ (15-17 years) experiences of receiving tactile massage and participating in the study?
• What were guardians’ experiences of their adolescents (15-17 years) receiving tactile massage, and their adolescents’ participation in the study?

This study used a process evaluation design with a primary emphasis on qualitative inquiry. The qualitative data consisted of interviews conducted separately with adolescents and guardians, exploring adolescents’ experiences of the intervention and guardians’ perceptions of their adolescents and any changes observed during the intervention. The qualitative data were complemented by descriptive quantitative questionnaire data completed by the adolescents, providing contextual information on their experiences, comfort, acceptability, and perceived feasibility of the intervention. An interactive approach was employed in which qualitative and quantitative components were integrated during interpretation, allowing for a comprehensive understanding of the intervention process. Quantitative data served a supportive role in relation to the qualitative findings, and integration occurred after separate analyses through result merging and discussion.

Interpretive description guided the qualitative component and constituted the main analytic focus of the study.^33^ Descriptive questionnaire data were collected concurrently to enrich and contextualise adolescents’ experiential accounts rather than to enable outcome comparisons. The Consolidated Criteria for Reporting Qualitative Research (COREQ)^34^ checklist was used to guide appraisal and reporting of the qualitative component. Given the study’s emphasis on experiential depth and integration, formal mixed methods reporting frameworks, such as the Mixed Methods Appraisal Tool (MMAT),^35^ were used as orienting references rather than as prescriptive templates.

### 2.2. Data Collection for Study 2

At baseline (T0), at least six weeks before the first session, adolescents completed the “*Access to Healthcare Questionnaire 5A”*. This questionnaire was repeated at follow-up (T4) (three months after the last massage session). After each of the ten massage sessions, participants anonymously submitted a “*Post-Massage Questionnaire”*. Two weeks after their last massage session (T3), qualitative interviews were conducted with adolescents and their guardians. Member checking in the form of follow-up interviews was conducted three years later (Jan–Feb 2025) with adolescents (n=9) and guardians (n=6).

### 2.3. Qualitative Data

Semi-structured individual interviews were conducted virtually via Zoom with the adolescents (n=13) and their guardians (n=12) approximately two weeks after the final massage session. The interviews with the adolescents were held by VN and SN from 05/2022 to 06/2022 and lasted 20-50 minutes (mean 29 minutes). Interviews with their guardians were held from 08/2022 to 09/2022 by the first author, (ACR) and lasted 20-50 minutes (mean 30 minutes). All interviews were audio recorded, transcribed verbatim and de-identified by assigning a letter to each adolescent from A to N.

The interviews were based on an interview guide with the opening questions: “Describe how it felt to receive a tactile massage” and “Describe your experience of being part of this study”. Other key questions included: “Describe your well-being and functioning before, during and after the study” and “Describe a time when you felt really dissatisfied, as well as really satisfied with the massage (a specific situation or in general)”. For the guardians, a semi-structured interview guide with a similar structure was used. The guide included opening questions: “Describe what it felt like when your child received tactile massage” and “Describe your experience of your child being part of this study”.

The first author (ACR) conducted the member checking. In total, 15 individuals participated: fourteen through interviews and one guardian through written feedback via email. A total of nine adolescents (six boys and three girls) and six guardians (guardians of 2 girls and 4 boys) participated. No guardians participated without a corresponding child. However, all interviews were conducted independently of each other, apart from two (one boy and one girl), which were held jointly with the adolescent and their mother. The interviews were conducted between 01/2025 and 02/2025. All interviews took place on Zoom, were audio recorded, and lasted between 20 minutes and one hour, with an average duration of 34 minutes.

### 2.4. Process Indicators/Acceptability and Feasibility Markers

#### 2.4.1. Adolescent Perspectives

Two questionnaires were developed by the research group to specifically address the research question from the adolescents’ perspectives, providing structured data to complement the qualitative data. The 5A questionnaire was constructed with items grounded in the conceptual framework of “Access to Health”.^36^ The post-massage questionnaire was constructed using a Visual Analogue Scale (VAS).^37^ These data were not used to test hypotheses or determine treatment effects but to support the interpretation of participants’ experiences.

#### 2.4.2. Access to Healthcare Questionnaire 5A

At baseline (T0), adolescents responded to five questions about their access, expectations, and acceptance of the intervention. These questions were slightly revised and repeated at T4. The questionnaire included both open-ended and closed-ended questions. Respondents could therefore choose to answer either in a brief and straightforward way (e.g. “yes,” “no,” “I don’t know”) or with more elaborate, narrative-style responses.

#### 2.4.3. Post-Massage Questionnaire

After each session, participants rated three aspects (expectations, comfort, and relaxation) on a 0–10 scale. To preserve anonymity, responses were sealed in envelopes and remained blinded to the massage provider and first author until after the study ended. Since the newly developed questionnaire was designed to describe the process as fairly as possible, questions were asked during data collection at T1, after the adolescents’ first massage session. The questions were: Does the questionnaire need to be adjusted? Is there anything important that should be included?

### 2.5. Data Analysis

#### 2.5.1. Procedures

Qualitative data were inductively analysed to illuminate experiences of tactile massage and study participation. Narrative answers from the “Access to Healthcare Questionnaire 5A” were included and integrated with the interview data in the qualitative analysis. Interviews with adolescents and caregivers were transcribed and coded using NVivo version 14. Analyses were guided by Interpretive Description, a practice-oriented approach suitable for applied health research, which allows for the thematic exploration of subjective experiences.^38^ Coding began broadly and was refined through constant comparison, identifying patterns, relationships, and latent content. Themes were developed iteratively by the first author (ACR) and the last author (SN), with team discussions conducted throughout the process to ensure analytical rigour. Within Interpretive Description, the process of participant feedback, described as member checking, serves to refine, deepen, and clarify the reflections and interpretations generated from the initial interview data. During the analysis, preliminary patterns and interpretations were shared with participants in follow-up conversations to explore whether the interpretations resonated with their experiences and to identify areas requiring clarification or further nuance. Participant reflections were incorporated iteratively into the ongoing analytic process. The first author (ACR) conducted these procedures and integrated the subsequent analysis with the findings from the original interviews. To strengthen trustworthiness, the coding structure and thematic framework were reviewed, discussed and refined by the full research team. Final themes were independently validated against the transcripts by ACR and SN to ensure congruence with the data.

#### 2.5.2. Analysis of Process Indicators/Acceptability and Feasibility Markers

Questionnaire data were analysed descriptively. Answers from the 5A questionnaire were coded to allow statistical processing. Since no fixed options were given, we created a coding system: 1 = Yes, 2 = No, 3 = I don’t know/Unclear/Both, to facilitate summarisation. Questionnaire responses are presented as medians and ranges, and categorical responses as frequencies where appropriate in Tables 2 and 3. Data were summarised in tables using STATISTICA v14.2 (2024), STAT Soft, Germany.

**Table 2.** Post-massage Questionnaire Scale Range 0–10, Where Higher Scores Indicate a More Positive Response. Frequency of Median = Number of Participants With the Median Value at Each Session. Participants A–L Completed 10 Tactile Massage Sessions

Tactile massage session	Median (range)	Frequency of median
**A. Was the massage what you expected?**		
1	8 (2-10)	4/12
2	10 (8-10)	9/12
3	10 (8-10)	9/12
4	10 (8-10)	8/11
5	10 (8-10)	8/11
6	10 (8-10)	11/11
7-10	10 (8-10)	8/10
**B. Did you feel at ease with the massage provider?**		
1	10 (8-10)	7/12
2	10 (9-10)	10/12
3	10 (9-10)	11/12
4	10 (8-10)	8/11
5	10 (8-10)	8/11
6	10 (9-10)	10/11
7-10	10 (8-10)	9/10
**C. Were you able to relax during the massage?**		
1	9 (8-10)	5/12
2	9 (7-10)	5/12
3	10 (7-10)	7/12
4	9 (6-10)	5/11
5	10 (9-10)	8/11
6	10 (7-10)	10/11
7-10	10 (8-10)	7/10

*Note.* Responses were missing for some later sessions. Data for participants M (6 sessions) and N (3 sessions) are presented separately in the results text.

**Table 3.** Access to Healthcare Questionnaire 5 A. The Five “A” Questions Were Administered Before the Intervention (T0) and Three Months After the Tactile Massage Intervention Ended (T4)

Time point	T0	T4	T0	T4	T0	T4
**Number of participants**	12^A-L^	​	1^M^	​	1^N^	​
**Number of conducted massage sessions**	10	​	6	​	3	​
1.*Was it easy for you to get here?*						
Positive	12	12	1	1	1	md
Negative	-	-	-	-	-	-
Unclear/both	-	-	-	-	-	-
2. *Are you clear about what you will be part of and what you will be doing? (Did…were…)*						
Positive	11	10	1	1	1	md
Negative	-	1^G^	-	-	-	-
Unclear/both	1^K^	1^L^	-	-	-	-
3. *Do you think it will be worth your time and energy to participate in the study? (Was…)*						
Positive	12	10	1	1	1	md
Negative	-	-	-	-	-	-
Unclear/both	-	-	-	-	-	-
4. *How do you think the massage provider will treat you during the massage? Will she be attentive to your needs?*						
Positive	12	10	1	1	1	md
Negative	-	-	-	-	-	-
Unclear/both	-	1^C^	-	-	-	-
5a. T0: *What do you think it will be like to get a tactile massage?*						
5b. T4: *Would you consider doing it again?*						
Positive	9	8	1	1	1	md
Negative	-	-	-	-	-	-
Unclear/both	3^G,I,L^	4^E,J,K,L^	​	​	​	​

*Note.* md = missing data. Participant cohorts and number of conducted tactile massage sessions: A–L = 10 sessions; M = 6 sessions; N = 3 sessions.

## 3. Results

### 3.1. Qualitative Results

Two central themes were constructed that capture the adolescents’ experiences with the tactile massage intervention, as well as the guardians’ perceptions:

1. The professional ritual in interaction within a safe environment
2. Touch as a pathway to calm and self-awareness

The member-checking process supported and confirmed the interpretations derived from the original interview data. Participants recognised the findings as consistent with their experiences and did not identify any substantive omissions, contradictions, or new themes. While some participants provided additional reflections and examples, these did not alter the existing analysis. Consequently, the integrated analysis remained consistent with the findings generated from the original interviews, with member checking serving to strengthen the credibility and resonance of the interpretations.

### 3.2. Theme 1: The Professional Ritual in Interaction Within a Safe Environment

Participants consistently described the massage experience as grounded in predictability, professionalism, and respectful interaction. A fixed treatment structure was a prerequisite for the ability to relax and experience psychological safety. The adolescents said that having a fixed treatment structure could be more important when experiencing neuropsychiatric challenges. The adolescents also reported that the repetition of familiar elements allowed them to feel oriented and in control, even when massage providers were replaced by others.

“One good thing, at least for me personally, was that there was music in the background and that it was the same playlist every time, so I recognised the songs… it became like an unwritten routine… and, that there weren't too many changes in the little things.” (Participant A, girl,16 years old)

The importance of clear communication, both before and during sessions, was emphasised. Providing text reminders before sessions with information about the time and who would be performing the massage, were considered valuable. The clarity that this gave appeared to ease the introduction to the massage sessions and reduce uncertainty. The massage providers’ practice of knocking before entering and leaving the room while participants changed was described as a sign of respect, helping to lessen feelings of vulnerability in an otherwise exposing situation. While some participants expressed a strong preference for massage provider continuity, others felt that the structured framework compensated for this. The physical environment, dim lighting, calm music, and towels also contributed to a feeling of comfort and safety.

“It was pretty calm and they worked on the same body part every time. They did it in a certain order all the time.” (Participant M, boy, 15 years old)

Adolescents’ experiences of massage treatment were influenced by external factors like school stress, family issues, and scheduling conflicts. Schoolwork could be delayed due to appointment times, especially when medication was active. Proximity to the treatment centre made attendance easier, while distance could be a challenge. Guardians supported participation by providing transport, reminders, and schedule adjustments. Overall, guardians felt that the effort was manageable and worthwhile.

### 3.3. Theme 2: Touch as a Pathway to Calm and Self-Awareness

The interviews highlighted that tactile massage, through a safe and gradual process, appeared to foster an experience of mental and physical calm. The adolescents described the sessions as “a break from the outside world”– a rare opportunity to rest both mentally and physically from an overstimulating daily life. Massage was also experienced as restorative, with many participants describing lingering effects lasting hours or days.

“I was tired all the time and very stressed… Yes, it has helped me a lot to relax and not be so stressed… I was able to take it easy and not be so stressed and not have to think about everything I had to do.” (Participant L, girl,16 years old)

Initial hesitance about physical touch due to shyness, unfamiliarity, or concern about exposure gave way to acceptance and appreciation over time. Feelings of embarrassment and discomfort, such as ticklishness during foot massage, concern about body odour or unfamiliarity with hand massage, gradually subsided after one or two sessions. Participants reported growing more comfortable with their bodies.

“I could freeze just from a light touch – but I don’t do that anymore. It has gotten much better after the massage. Now, for example, I can have the cats on my lap, and it feels much easier to handle other forms of touch.” (Participant D, boy,17 years old)

Participants described a variety of experiences, from a sense of calm and recovery in the moment to increased body awareness and the ability to recognise stress signals in the longer term. Stress was described as vague or difficult to distinguish from hyperactivity. The calm experienced during massage sessions served as a reference point, helping them identify and regulate both emotional and physical states. The adolescents stated that the massage allowed them to experience genuine relaxation for the first time. It had also given them access to previously unknown physical or emotional experiences.

“Let’s say that I feel a little better about myself and know when I'm stressed and (know) where the boundaries are, like… that I can sometimes stop and just take it easy for a while… It’s helped me understand that it’s good to take it easy sometimes.” (Participant I, boy, 17 years old)

Guardians experienced that their adolescents became more independent in that they began to handle their own appointments. Their willingness to engage in something unfamiliar was seen as a sign of maturity and boosted self-esteem. The massage was also seen as a valuable form of recovery, especially for those struggling with school or daily life, offering a fresh starting point. Guardians noted that the treatment seemed to help their children “land” after high activity or stress, creating space for reflection and emotional reset.

“He can wear himself out physically, but mentally he’s still running at full speed. But I feel that this massage calmed him down, even mentally… It’s a good kind of tiredness… And that he was able to slow down and didn’t need to come up with things to do, or go out, or go to the gym. He was content. He had landed.” (Guardian of participant F, boy 17 years old)

### 3.4. Results of Process Indicators/Acceptability and Feasibility Markers

At T0, data from all 14 participants were analysed. At T4, participant N’s data were unavailable as he withdrew from the study after attending three massage sessions. Statistics on massage satisfaction showed significant differences between the first and subsequent sessions for all participants involved in the study.

### 3.5. Post-Massage Questionnaire

As shown in Table 2, post-massage questionnaire responses indicated high levels of acceptability and feasibility throughout the intervention. Participants generally reported increasing relaxation over the course of the study, with relaxation ratings tending to be higher in later sessions than during the initial session. Satisfaction with the massage experience was also consistently high. Although satisfaction ratings after the first session varied (ranging from 2 to 10), most participants assigned the highest possible rating from the second session onward, suggesting that the intervention met or exceeded expectations. Participants also reported feeling safe and comfortable with the massage provider throughout the intervention period. Ratings for safety and comfort remained consistently high, ranging from 8 to 10 across sessions. Notably, responses to all three questionnaire items were similar across the seven massage providers, indicating a consistent participant experience regardless of massage provider and supporting the overall acceptability and feasibility of the intervention.

Participants M (6 sessions) and N (3 sessions) reported a similar pattern to the main cohort. For Question 1, participant M scored 8 at session 1 and 10 at sessions 2–6, whereas participant N scored 7 at session 1 and 10 at sessions 2–3. For Question 2, participant M scored 9 at session 1 and 10 thereafter, while participant N scored 10 at all completed sessions. For Question 3, participant M scored 8, 7, 9, 10, 10, and 10 across sessions 1–6, whereas participant N scored 8, 7, and 9 across sessions 1–3.

### 3.6. Access to Healthcare Questionnaire 5A

Results from the 5A questionnaire supported the trends in the Post-Massage Questionnaire. All five domains showed clear positive development from baseline (T0) to post-intervention (T4). Nearly all participants found the study worthwhile, felt their needs were met, and expressed willingness to receive massage again or join similar studies in the future.

## 4. Discussion

This study illuminated adolescents’ and guardians’ experiences of ten structured tactile massage sessions characterised by safety, trust, and predictability. As one of the first studies to examine tactile massage in adolescents with ADHD, it addressed a gap in understanding how affective touch is experienced in this population. The findings showed that tactile massage was feasible and acceptable, supported calm and relaxation, increased bodily awareness, and fostered embodied interconnectedness through integrated bodily, emotional, and relational experiences.

### 4.1. Integration of the Results

**Figure 2.**
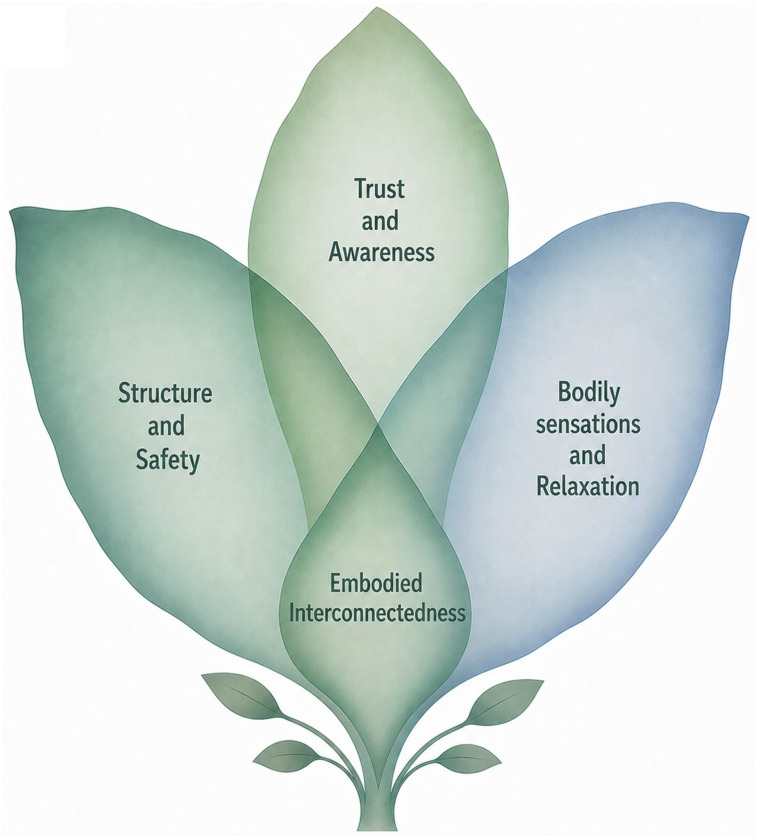
Conceptual model of embodied interconnectedness as an interpretive lens for tactile massage, showing how trust and awareness, acceptability and relaxation, as well as structure and safety contributed to adolescents’ overall sense of embodied interconnectedness

As illustrated by the participants’ narratives in the results, Theme 1 (the professional ritual) provided the relational foundation, while Theme 2 (touch as a pathway) facilitated the internal shift. Adolescents described changes in awareness of internal bodily states alongside calm and security in interactions with others, suggesting a shift toward improved body–emotion alignment. Together, trust, self-regulation, and emotional comfort reflected an integrative internal process. Quantitative process indicators showed increased relaxation and satisfaction over time, reinforcing the richer qualitative accounts of gradual changes in relaxation and comfort. The structured intervention, characterised by predictability, respectful interaction, and affective touch, appeared to create conditions in which embodied interconnectedness could develop and become internalised. Adolescents described the sessions as a rare opportunity for restorative stillness, marked by attentiveness, emotional availability, and enhanced capacity to interpret and integrate sensory experience. These experiences extended beyond immediate relaxation and included shifts in bodily awareness, emotional orientation, and a growing sense of inner coherence, with perceived carry-over influences into daily life Figure 2.

Conceptual model of embodied interconnectedness as an interpretive lens for tactile massage, showing how trust and awareness, acceptability and relaxation, as well as structure and safety contributed to adolescents’ overall sense of embodied interconnectedness.

### 4.2. Embodied Interconnectedness as an Interpretive Lens for Therapeutic Touch

The findings emphasise that therapeutic touch is effective only when essential elements support embodied interconnectedness. CT-targeted touch represents a novel, precision-based approach, with tactile massage providing a practical means of delivery. For adolescents with adhd, structured, sensory-rich interventions may enhance engagement, emotional regulation, and receptivity, demonstrating transformative potential for personalised paediatric and adolescent mental health care.

Embodied interconnectedness is conceptualised as an interpretive lens through which a dynamic, embodied sense of relation to oneself, others, and one’s lived body is understood. The findings indicate that adolescents experienced increased physical, emotional, and relational coherence through the intervention. These results are relevant to psychiatric mental health nursing because they highlight embodied interconnectedness as an often-overlooked aspect of care and show how touch, as a caring act, can create an integrated sense of physical and mental cohesion. This perspective is especially important since traditional psychiatric treatments do not always meet the adolescents’ embodied and relational needs. Embodied interconnectedness strengthens the understanding of the care relationship as a dynamic and relational process, where connections between people, bodies, and the environment form a foundation for health. From this viewpoint, nurses and other massage providers can use structured, body-based care grounded in predictability, respectful interaction, and relational safety to support emotional regulation, build trust, and enhance embodied self-awareness, regardless of the specific massage techniques used. Thus, embodied interconnectedness could validate the role of the nurse as a facilitator of the patient’s integrated sense of physical and mental cohesion, ensuring that the often-overlooked embodied needs of neurodivergent adolescents are met.^30^

Trust, self-regulation, and bodily awareness functioned as key subdimensions, reflecting greater awareness of internal states and stronger relational engagement. The results from Theme 1 (The professional ritual in interaction within a safe environment) support the relational dimension of embodied interconnectedness. Adolescents emphasised that the “fixed treatment structure” and “repetition of familiar elements” were prerequisites for psychological safety. Quantitative data showed that participants felt consistently “at ease with the massage provider” (median 10/10), regardless of which of the seven massage providers performed the session. This indicates that the ritual itself, rather than just the individual massage provider, provided a secure ‘meeting place’, as also shown in previous research.^20^ Thus, these effects were not only experienced as influences of treatment but as relational qualities made possible through a predictable, respectful, and non-intrusive therapeutic environment. The structured massage sessions provided adolescents with a stable and predictable space that supported calm sensory processing and growing agency. These findings align with prior research showing that clearly structured therapeutic rituals and well-defined interpersonal boundaries enhance comfort, emotional receptivity and engagement.^39,40^ Sensory and relational elements can help ease heightened alertness and emotional detachment in adolescents with neuropsychiatric conditions. Structured, safe physical contact within a professional therapeutic framework promotes embodiment and facilitates emotional connection.^20^ In theme 2 (Touch as a pathway to calm and self-awareness) the participants described a transition from initial “hesitance” or “freezing” to a state of “acceptance and appreciation” of touch. Consistent with prior research, such rituals may offer adolescents a somatic reference point for calm – a physiological memory they can access during stress.^41,42^ Consistency and structure in the therapeutic environment could be especially important for adolescents with adhd, providing a scaffold that can support a sense of calm and sustained participation.^40,43^ A predictable sensory and relational framework may contribute to gradual adjustment and everyday coping.^39^

Viewing tactile massage through the lens of embodied interconnectedness offers a helpful way to understand how sensory-based interventions can support growth and well-being in neurodivergent adolescents. This supports theories of interoceptive development and suggests tactile massage may play a role in modulating sensory regulation and addressing interoceptive dysfunction – a significant challenge for adolescents with adhd.^3,44,45^ Difficulty noticing internal stress signals is associated with emotional outbursts, problems with self-soothing, and bodily discomfort,^46-48^ suggesting a need for interventions that help build awareness of bodily signals. Being more aware of bodily signals is linked to better emotion regulation, decision-making, and emotional endurance.^49^ This perspective shifts the focus from behaviour and cognition to the role of the body and relational safety in supporting well-being. The findings may help guide the design of integrative, conceptually grounded approaches that prioritise meaningful and relational engagement with the body as a basis for well-being. The overall experience for participants was characterised by “restorative stillness” and a sense of “landing”. Guardians observed that their children, who were often “mentally running at full speed”, were able to slow down and find a state of physical and mental cohesion. This experience of wholeness is central to the concept of embodied interconnectedness. It suggests that tactile massage offers more than transient relaxation; it facilitates a move toward an integrated sense of self where physical relaxation, emotional regulation, and relational security reinforce each other. One example is the growth in agency and independence reported by both adolescents and their guardians, suggesting that embodied experiences can support positive changes in everyday functioning.^50^ This aligns with research showing that adolescents’ ability to manage emotional challenges through self-awareness and coping strategies is closely linked to autonomy and a sense of competence.^51^

### 4.3. Strengths and Limitations

A strength of this study lies in its qualitative depth and the inclusion of both adolescent and guardian perspectives, enabling a nuanced understanding of experiences within a real-world psychiatric setting. The use of interpretive description supported the generation of clinically relevant insights grounded in participants’ narratives, while longitudinal member checking further strengthened the credibility and richness of the data.

Limitations include the small, culturally homogeneous, and geographically localised sample, as well as the exploratory nature and use of non-validated descriptive questionnaire measures. Consequently, the findings cannot be generalised beyond similar contexts. However, this emphasis on depth over breadth was intentional, allowing for close examination of care processes rather than estimation of effect sizes. Quantitative data were therefore used descriptively to contextualise qualitative findings and served a confirmatory rather than explanatory role. The qualitative findings provided a robust interpretive basis for understanding therapeutic processes and for developing an overarching conceptual model.

## 5. Conclusion

This study contributes to the limited and emerging research on affective touch interventions in adolescents with adhd. By linking adolescents’ lived experiences with CT-fibre research, the findings offer initial insight into how affective touch therapy may be experienced and understood in a clinical context. Tactile massage was experienced by adolescents with adhd as a feasible, acceptable, and calming caring encounter within child and adolescent psychiatric services. Participants described experiences of bodily awareness, emotional calm, and relational safety, which are interpreted through the lens of embodied interconnectedness. These findings suggest that structured, sensory-informed interventions may offer everyday support for adolescents with adhd. The structured ritual described by the participants mirrors established nursing principles of consistency, ethical boundaries, and attentiveness to vulnerability. The concept of embodied interconnectedness may help bridge emerging knowledge from CT-touch research with the lived experiences of adolescents with adhd, receiving tactile massage in clinical care.

## 6. Future Research

Future research should examine embodied interconnectedness as a conceptual and measurable outcome, using diverse samples and controlled designs. Research employing longitudinal and comparative designs might explore how embodied, sensory-based interventions and experiences of embodied interconnectedness may be integrated within multimodal adhd care across more diverse populations and settings. The potential of tactile massage to offer accessible, structured, and relationally safe therapeutic experiences merits further clinical exploration. With adhd diagnoses rising in Sweden and internationally,^52,53^ early interventions targeting hyperactivity, inattention, and impulsivity are increasingly important. Based on current findings, tactile massage could serve as a potentially supportive early intervention.^28,31^ Embodied interconnectedness supports a deeper understanding of the care relationship as a dynamic and integrated process. This points to a need to further develop nursing science concepts that include the embodied dimension of care to a greater extent.

## 7. Implications for Practice

By identifying relational and contextual conditions that support the implementation of CT-based touch interventions, this study contributes to bridging the gap between experimental research on affective touch and its application in everyday clinical practice. For psychiatric and mental health nursing, the findings suggest that predictable, respectful, and sensory-informed caring encounters are of relevance. Tactile massage functioned not primarily as a technique, but as a structured caring relationship characterised by attentiveness, professionalism, and ethical sensitivity. Incorporating such embodied approaches may complement existing interventions by offering adolescents alternative ways to experience calm, safety, and bodily awareness within care. As tactile massage did not rely on verbal insight or cognitive capacity, it was accessible to adolescents who struggle to engage in traditional talk-based therapies. The concept of embodied interconnectedness describes how touch can create an experience of wholeness, where physical relaxation, emotional regulation and relational security are experienced simultaneously and reinforce each other. In this context, tactile massage appears not only as a method or intervention but also as a caring meeting place where physical presence and relational security intersect.

## Supplemental Material

Supplemental Material - Introducing the Concept of Embodied Interconnectedness: Perspectives on Tactile Massage for Adolescents With Attention Deficit Hyperactivity Disorder (Adhd) Supplemental Material for Introducing the Concept of Embodied Interconnectedness: Perspectives on Tactile Massage for Adolescents With Attention Deficit Hyperactivity Disorder (Adhd) by Anna-Carin Robertz, Anne-Katrin Kantzer, Viola Nyman, Carl-Johan Törnhage and Stefan Nilsson in Global Advances in Integrative Medicine and Health.

## Data Availability

The quantitative datasets used and analysed during the current study are available from the corresponding author at reasonable request. The qualitative dataset generated and analysed is not publicly available due to ethics approval for the study, which requires that the transcribed interviews be kept in locked files, accessible only to the researchers.*
