# Supplementary material for: Introducing the Concept of Embodied Interconnectedness: Perspectives on Tactile Massage for Adolescents With Attention Deficit Hyperactivity Disorder (adhd)
Source: Glob Adv Integr Med Health. 2026 Aug 3;15:27536130261475332. doi: 10.1177/27536130261475332 (PMC13438357; doi:10.1177/27536130261475332)
Supplement: Supplementary file 1 [file sj-pdf-1-gam-10.1177_27536130261475332.pdf]

Supplementary file S1: Protocol for the Tactile Massage (TaktiPro®) Sessions

Quality and Consistency

- All massages were performed by certified TaktiPro® therapists who had previously completed two days of theory and practice, followed by 60 hours of supervised hands-on training and passed both theoretical and practical examinations.
- Therapists strictly followed the established protocol, using predetermined and structured movements.
- Regular supervision and ongoing communication ensured adherence to the protocol.
- Therapists were not involved in data collection or analysis. The first author (XX) collected post-massage questionnaires but did not review them until the intervention was completed.
- For each session, therapists followed a checklist covering:
    - **Health and safety measures** (e.g. symptom checks and use of protective equipment, particularly during the COVID-19 startup phase)
    - **Administrative tasks** (e.g. managing questionnaires, schedules, and supplies)
    - **Communication with XX** (e.g. sick notifications, schedule inquiries, intervention updates, and supply requests)
    - **Follow-up practices** (e.g. offering refreshments, providing reminders, and recording reflective notes)

STEP 1: Installation of the participant (~ 5 min)

- The massage room is prepared with dim lighting and soft background music (the same music used for all sessions).
- Unscented vegetable oil is prepared for the massage.
- The adolescent is invited to lie down on a massage couch prepared with a clean sheet and pillows.
- The therapist leaves the room while the adolescent settles in, re-entering only after knocking.
- A blanket is placed over the participant to maintain comfort and privacy; hands and feet are covered in small towels.

STEP 2: Contact and preparation (~ 2 min)

- The therapist briefly explains the procedure and ensures the participant is comfortable.
- A small amount of unscented vegetable oil is warmed between the therapist's palms.
- Gentle contact is established by lightly placing both hands on the participant's shoulders over the blanket. The area to be massaged (back) is then uncovered.

Step 3: Tactile Massage Session (~45 min)

1. **Back massage (~10 min.)**
    - The adolescent lies prone, covered with a blanket except for the back.
    - Slow, circular and transverse strokes are applied using the palms and fingers together.
    - Movements are soft yet firm, covering the width and length of the back.
    - Gentle sliding strokes are repeated in a slow, regular rhythm.
    - Both palms are placed flat on the back, performing wide circular strokes with even pressure.
2. **Arms and hands (~12 min.)**

    - The therapist holds the blanket to allow the adolescent to turn safely onto their back without exposure.
    - One arm is uncovered.
    - Circular and lengthwise strokes are applied with the full palm along the arm.
    - The hand is massaged with gentle circular strokes on the palm.
    - Each finger is lightly stretched and rotated individually before the arm is repositioned under the blanket
3. **Feet (~10 min.)**
    - The adolescent lies supine with one foot uncovered.
    - The therapist holds the heel with one hand while massaging the sole and top of the foot with the other.
    - Circular movements are applied around the ankle joint.
    - Each toe is gently massaged with light traction and small circular motions.
    - The foot is then covered before proceeding to the other side.
4. **Integrative stroke (~3 min.)**
    - The adolescent lies supine, fully covered by the blanket.
    - The therapist stands at the head of the couch.
    - Both palms glide continuously from the crown of the head to the soles of the feet.
    - This stroke is repeated three times in a slow, uninterrupted motion, symbolising closure and integration of the session.

STEP 4: Concluding procedures (5 min.)

- A brief period of quiet rest is allowed before the participant is invited to sit up slowly and dress. The therapist leaves the room during this time.
- After knocking, the therapist re-enters, thanks the participant, and offers a light snack (options include non-carbonated juice, still or carbonated water, filled crispbread sandwiches, chocolate balls, or fruit).
- The adolescent completes the post-massage questionnaire, seals it in an envelope, and hands it to the therapist, who places it in a closed box.
- The therapist performs a nonverbal observational check to ensure the participant feels stable and comfortable before departure.

Duration: ~60 minutes per session (including installation, massage, and conclusion).
